# Thrombolytic Potential of Novel Thiol-Dependent Fibrinolytic Protease from *Bacillus cereus* RSA1

**DOI:** 10.3390/biom10010003

**Published:** 2019-12-18

**Authors:** Chhavi Sharma, Gad Elsayed Mohamed Salem, Neha Sharma, Prerna Gautam, Rajni Singh

**Affiliations:** 1 Amity Institute of Microbial Biotechnology, Amity University Uttar Pradesh, Noida 201313, India; sharmachhavi27@gmail.com (C.S.);; 2 National Organization for Drug Control and Research, 51 Wezaret El-Zeraa st., Giza 12618, Egypt

**Keywords:** fibrinolytic protease, *Bacillus cereus* RSA1, thiol-dependent, thrombolytic potential

## Abstract

The present study demonstrates the production and thrombolytic potential of a novel thermostable thiol-dependent fibrinolytic protease by *Bacillus cereus* RSA1. Statistical optimization of different parameters was accomplished with Plackett–Burman design and validated further by central composite design with 30.75 U/mL protease production. Precipitation and chromatographic approaches resulted in 33.11% recovery with 2.32-fold purification. The molecular weight of fibrinolytic protease was 40 KDa and it exhibited a broad temperature and pH stability range of 20–80 °C and pH 5–10 with utmost activity at 50 °C and pH 8, respectively. The protease retained its fibrinolytic activity in organic solvents and enhanced the activity in solutions with divalent cations (Mn^2+^, Zn^2+^, and Cu^2+^). The enzyme kinetics revealed K_m_ and V_max_ values of 1.093 mg/mL and 52.39 µg/mL/min, respectively, indicating higher affinity of fibrinolytic activity towards fibrin. Also, complete inhibition of fibrinolytic activity with DFP and a 2-fold increase with DTT and β-mercaptoethanol indicates its thiol-dependent serine protease nature. MALDI–TOF analysis showed 56% amino acid sequence homology with Subtilisin NAT OS = *Bacillus subtilis* subsp. natto. The fibrinolysis activity was compared with a commercial thrombolytic agent for its therapeutic applicability, and fibrinolytic protease was found highly significant with absolute blood clot dissolution within 4 h in in vitro conditions. The isolated fibrinolytic protease of *Bacillus cereus* RSA1 is novel and different from other known fibrinolytic proteases with high stability and efficacy, which might have wide medicinal and industrial application as a thrombolytic agent and in blood stain removal, respectively.

## 1. Introduction

Cardiovascular diseases (CVDs) have emanated as one of the leading causes for increase in mortality rate, and account for 31% of deaths world-wide [1]. The American Heart Association delineated that CVDs are responsible for more than 17.9 million deaths per year that shall exceed 23.6 million by 2030 [2]. The incongruity in the level of fibrin formation and fibrinolysis is responsible for the occurrence of cardiovascular diseases [3]. This medical condition, resulting in intravascular clotting in blood vessels, is termed as thrombosis [4,5]. These clots limit the flow of blood through veins and arteries, leading to cardiac ailments such as embolism, myocardial infarction, heart stroke, etc. [6,7,8,9]. Thrombin and Factor Xa (FXa) are identified as imperative components of blood clotting cascade [10]. The prothrombinase complex constitutes key component FXa, comprised of phospholipids, calcium ions, and factor Va [11]. This complex finally transforms prothrombin (inactive protein) into thrombin (active protease), converting soluble fibrinogen (glycoprotein) into fibrin polymer (insoluble blood clot) [5]. Fibrin clots are hydrolyzed by plasmin [12], which is stimulated from plasminogen by plasminogen activators (PAs) [13,14]. This natural dynamic equilibrium is disturbed when the process of natural fibrin clot hydrolysis undergoes pathophysiological shambles, leading to formation of fibrin clots. Such clots may cause hindrance in the circulation of blood, causing blockage in blood vessels, ultimately leading to cardiac disorders which are life-threatening [15,16].

Approaches such as use of anti-coagulant agents, anti-platelet drugs, fibrinolytic enzymes, and surgical operations are employed for the treatment of thrombosis and to dissolve the blood clots [17]. Further, there are numerous side effects which may occur following the administration of the available anti-thrombotic strategies, as well as the high expense, which limit their scope. The effects of reperfusion, urticaria (allergic reaction), and hemorrhage are the major inimical consequences of such thrombolytic approaches on human health [18,19]. Other after effects include headache, dizziness, ulcers, increased clotting time, nausea and vomiting, etc. Management of thrombosis using enzyme therapies in the form of urokinase type plasminogen activator, bacterial plasminogen activator, and tissue type plasminogen, etc., are widely practiced, but with low specificity and affinity for fibrin [20,21].

Nowadays, microbial fibrinolytic enzymes play a pivotal role in the management of cardiovascular disorders and have the ability to effectively dissolve blood clots with no after effects. Fibrinolytic enzymes are generally proteases which catalyze hydrolysis of proteins acting on the peptide bonds [22]. They can be easily produced on large scale with short generation time and are less expensive [23,24,25]. Fibrinolytic proteases basically belong to either the metalloproteases or the serine class of protease [26]. Numerous microbial fibrinolytic enzymes have been isolated and characterized from bacterial species, such as *Bacillus polymyxa* and *B. subtilis* [27], *E. coli* [28], *Pseudomonas* [29], *B. cereus* IND1 [30], *Streptomyces* [31], *Paenibacillus* sp. IND8 [32], *Serratia* sp. [33], and *Streptomyces rubiginosus* [34]. Various fungi have also been reported aiding in the production of such enzymes, such as *Penicillium chrysogenum* [35], *Fusarium* sp. [36,37], *Aspergillus* sp. [38,39], *Rhizopus chinensis* [40], and *Cordyceps militaris* [41].

The management of thrombosis using existing enzyme therapies of microbial origin have low blood clot dissolution rate. Thus, the quintessential pursuit for enhanced production of fibrinolytic protease with high efficacy has driven the statistical optimization of enzyme production. Generally, enzyme production is significantly influenced by medium components, such as nitrogen and carbon sources, and environmental aspects, such as temperature, inoculum, incubation time, and pH [42,43]. The analyses of effect of numerous physico-chemical parameters require a lot of experimentation and consumption of chemicals and time. Hence, Plackett–Burman Design and response surface methodology are applied for the enzyme production process to select the optimal levels of these parameters [30,44].

Plackett–Burman design is an extensively used statistical tool for screening of numerous parameters available to select the most profound ones [45]. This is followed by response surface methodology, which aids in determining the optimal level of the key variables involved in enhanced production of fibrinolytic protease [46]. These methods assist in reducing the number of experiments, time, and cost involved, and thus make the use of statistical techniques prominent tasks for the improved production of enzymes. 

Therefore, our study focused on the production of a novel fibrinolytic protease with high anti-thrombotic efficacy from *Bacillus cereus* RSA1. This research showed that the purified fibrinolytic protease has high stability within a wide range of temperatures and pH levels, along with the solutions of organic solvents, metal ions, and inhibitors/activators. Also, the anti-thrombotic effects of fibrinolytic protease enabled drastic blood clot dissolution when analyzed with a commercial drug and laboratory protease. As per our knowledge, this is one of the few research reports wherein an efficient thiol-dependent novel serine fibrinolytic protease has been obtained, which might have wide applicability on the medicinal and industrial fronts as a thrombolytic and blood stain removal agent, respectively. In summation, we reveal fibrinolytic protease with high efficacy, specificity, and stability, suggesting it to be a novel and potent fibrinolytic protease.

## 2. Materials and Methods

### 2.1. Isolation and Screening of Bacterial Strain Producing Fibrinolytic Protease

The isolation of 400 bacterial strains was accomplished from the soil samples collected from junk heaps fetched from (15 cm depth) numerous dumping sites of Noida (U.P, India). The procured soil samples were collected in sterile polyethylene bags. The samples were then diluted serially in sterile saline solution (0.9% NaCl). Up to 1 × 10^−5^ g/mL. 100 μL of each aliquot was spread on the sterile nutrient agar plates and were incubated at 37 °C for 48 h. The isolates were then purified and gram characterized for initial identification.

The isolates with different morphological characteristics were selected and cultivated on sterile skim milk agar plates (powdered skimmed milk 0.5% and agar 2.0%) with incubation for 48 h at 37 °C. After incubation, the potent isolates in proteolytic activity (based on zone diameter around the colony) were selected for the subsequent studies. Stock cultures of the strains were preserved at −20 °C in glycerol solution until further use.

### 2.2. Fibrinolytic Activity of the Screened Strains

Fibrinolytic activity of the selected strains was examined in a fibrin plate comprised of 1.2% fibrinogen, thrombin (100 NIH units/mL), 1.0% agarose, and 0.1 M Na_3_PO_4_ buffer (pH 7.5). The strains showing a clear zone around the colony streak indicate their fibrinolytic protease producing quality [47]. Three strains showing the fibrinolytic activity were retained for further study. The potent isolate was then subjected to identification through biochemical characterization and 16S rDNA sequencing.

### 2.3. Identification of the Microbial Strain

The biochemical and morphological characteristics of the microbial strain were evaluated as illustrated in the Bergey’s brochure for identification of bacteriology. The strain was further identified using 16S rDNA sequencing. Extraction of genomic DNA of the strain was accomplished using a modified phenol–chloroform extraction method [48]. Amplification reaction was performed in a 50 µL PCR vial by mixing 1 µL template DNA, 4 µL (75 pmol/µL) forward primer (5′ CAGCAGCCGCGGTAATAC 3′), 4 µL (75 pmol/µL) reverse primer (5′ TACGGCTACCTTGTTACG 3′), 25 µL master mix (1X, G-Biosciences) containing Taq polymerase, and dNTPs and PCR reaction buffer. DNA amplification was performed in a DNA Thermal Cycler (Master cycler pro, Eppendorf) following the underneath mentioned profile: Initial denaturation at 95 °C for 5 min, 30 cycles of denaturation (94 °C for 30 s), annealing (50 °C for 30 s), and extension (72 °C for 1 min), with a final extension at 72 °C for 5 min. The amplified product with molecular weight of DNA markers was run on a 1.0% agarose gel mixed with EtBr (0.5 µg/mL) at persistent voltage (60 V) and visualized in gel documentation system (InGenius3, Syngene). The purification of the amplified DNA product from the gel was accomplished using Qiagen gel elution kit as per the manufacturer’s instructions and protocol. The pure eluted amplified DNA product was sent to sequencing [49].

### 2.4. Phylogenetic Analysis and Strain Identification 

The obtained 16S *r*DNA sequence was subjected to nucleotide blast (blastn) at NCBI to retrieve homologous sequences and identify the strain to the generic level. The multiple sequences were aligned using CLUSTALW2 (EMBL-EBI, Hinxton, Cambridgeshire, UK), and the phylogenetic tree was created through the neighbor-joining method in Phylip and viewed using Tree View program (1.6.6, Glasgow, Scotland, UK) [50].

### 2.5. Fibrinolytic Protease Production

Fermentation was initially carried out in elementary flasks (100 mL) filled with production media (50 mL), prepared by reviewing previously cited literature which comprised of (g/L) glucose (10.0), soybean (10.0), CaCl_2_ (0.5), MgSO_4_·7H_2_O (0.5), NaCl_2_ (0.5), and K_2_HPO_4_ (5.0). The flasks were inoculated with 1% of the inoculum and incubated at 37 °C for 24 h at 120 rpm in a shaker incubator. The fermentation broth was centrifuged at 10,000 rpm for 10 min [49], and fibrinolytic protease activity was assessed using supernatant as crude enzyme.

### 2.6. Activity Assay of the Fibrinolytic Protease Produced

The activity of fibrinolytic protease produced in the initial media was detected using fibrin as substrate [44]. The free amino acids (tyrosine) liberated were determined using Folin–Ciocalteu’s phenol reagent, and fibrinolytic activity of the protease was evaluated through standard curve of tyrosine. Fibrinolytic protease activity (one unit) was well-précised as the quantity of enzyme essential to liberate 1 µg/mL tyrosine per minute [5].

### 2.7. Optimization of Media

Optimization of the production media using experiment statistical design was conducted for the increase in production of fibrinolytic enzyme and was done using two methods. In the first method, screening of variables was done through Plackett–Burman design. The second method encompassed the optimization of substantial variables by the response surface method, employing the central composite design (CCD). Design Expert^®^10.0.8.0 (Stat-Ease Inc., Minneapolis, MN, USA) was used to design and analyze the experimental data.

### 2.8. Elucidation of Significant Variables

Plackett–Burman design was used for quick screening of numerous factors to discover the most important independent components [45,51]. The impact of independent variables of the enzyme production such as peptone (A), yeast extract (B), casein (C), glucose (D), sucrose (E), initial pH (J), K_2_HPO_4_ (F), MgSO_4_ (G), inoculum size (L), speed of agitation (H), and incubation time (K) was studied. Individual factors were tested at both low (−1) and high (+1) level. Then, the eleven factors were screened with 13-run Plackett–Burman design. The influence of each variable was diagnosed using Equation (1):

E(x_i_): 2(ƩM_i_^+^ − ƩM_i_^−^)/N (1)

where E(x_i_) signifies the outcome of the variables, M_i_^+^ and M_i_^−^ are the total production of the enzyme where the measured variable (x_i_) was existing at both low and high concentrations, and N denotes the total number of trials.

### 2.9. Central Composition Design

The determination of the optimal level of important factors screened through Plackett–Burman design was performed through response surface methodology using CCD. Four independent selected factors (peptone, yeast extract, casein, and glucose) were analyzed for improving the production of protease. These factors were examined at three diverse stages (−1, 0, +1). A total number of 30 trials were executed in duplicates. The data attained from CCD were analyzed by ANOVA (analysis of variance). A second-order quadratic function was used to denote the function of the interacting factors to calculate the predicted response Equation (2):

Y = β_0_ + β_1_X_1_ + β_2_X_2_ + β_3_X_3_ + β_11_X_1_^2^ + β_22_X_2_^2^ + β_33_X_3_^2^ + β_12_X_1_X_2_ + β_13_X_1_X_3_ + β_23_X_2_X_3_ (2)

where Y is response; X1–X3 are independent variables; β0 is the intercept; β1–β3 are linear coefficients; β11, β22, and β33 are quadratic coefficients; and β12, β13, and β23 are interaction coefficients, respectively. 

The model function was denoted in the form of three-dimensional (3D) contour plots which expressed the relationship between the varied levels of each test variable and the subsequent response generated. Validation of the model was done using the significant variables under conditions, as predicted by the CCD.

A random set of five experimental combinations was set up to verify the recommendations of the model. The experiments were done in triplicates for authenticating the designed model.

### 2.10. Purification of Fibrinolytic Protease

Fermented culture of microbial biomass was harvested and centrifuged at 8000 rpm for 20 min at 4 °C. The cell debris was disposed of and the supernatant was used for purification. The cell extract proteins were precipitated with chilled ethanol (1:2 proportion) and incubated overnight at −20 °C. The proteins obtained in the form of precipitates were procured by centrifugation (8000 rpm for 10 min). The pelleted proteins were dissolved in 50 mM phosphate buffer (pH 7.5) and applied on a pre-equilibrated (50 mM Tris HCL buffer, pH 7.5) Sephadex-G75 column (50 × 15 mm; Sigma Aldrich, St. Louis, MO, USA) for purification. The protein was eluted at a flow rate of 1.0 mL/min with the same buffer, and collected fractions were examined for fibrinolytic activity. The fractions with enzymatic activity were pooled, lyophilized, and assayed by SDS-PAGE. 

### 2.11. Diagnosis of Amino Acid Composition of Fibrinolytic Protease Using MALDI Mass Spectrometry

The purified fibrinolytic protease bands obtained through SDS-PAGE were excised and in-gel trypsin digested. The peptides obtained were then mixed with matrix HCCA (α-Cyano-4-hydroxycinnamic acid) in 1:1 ratio. The determination of the amino acid composition was accomplished through MALDI-TOF mass spectrometric analysis (MALDI-TOF/TOF MS Bruker Daltonics ULTRAFLEX III), followed by MS/MS of the respected peptides. Further analysis was done with FLEX ANALYSIS SOFTWARE for recording the MS and MS/MS spectrum. The masses obtained via fragmentation of the peptides in MS and MS/MS were submitted for MASCOT search against the given sequence for significant identification of the protein. The recognized peptide values were put through BLAST search in NCBInr for Swissprot protein sequences and the Protein Data Bank proteins against proteins of *Bacillus* sp. origin. The blastp algorithm in the NCBI databases was employed for the study (http://blast.ncbi.nlm.nih.gov/Blast.cgi).

### 2.12. Characterization of the Purified Fibrinolytic Protease

#### 2.12.1. Optimization of Temperature and pH for Maximum Fibrinolytic Protease Activity

The evaluation of maximum protease activity was assayed at temperature range of 20–80 °C after 10 min of incubation with the fibrin as substrate. The effect of pH on protease activity was estimated by enzymatic assay with buffers ranging from pH 5.0 to 10.0 [52].

#### 2.12.2. Stability of Fibrinolytic Protease at Different Temperature and pH Range

The thermal constancy of the protease was evaluated by incubating the enzyme at temperature range 20–80 °C for 2 h without the fibrin (substrate) fractions. The stability of protease was evaluated by incubating the enzyme in different buffers (pH 5.0–10.0) without the substrate for 2 h. Enzyme samples were withdrawn and assayed for their temperature and pH stability at every 30, 60, 90, and 120 min intervals [52].

#### 2.12.3. Kinetic Studies

The kinetic parameters K_m_ and V_max_ of the protease were estimated by non-linear regression, Michaelis–Menten curve using GraphPad Prism 8.Ink with various concentrations (0.2–2 mg/mL) of fibrin as substrate [53].

#### 2.12.4. Effect of Different Metal Ions, Inhibitors/Activators, and Organic Solvents on the Fibrinolytic Protease Activity

The enzyme was incubated for 1 h at 37 °C with 2 mM and 5 mM concentration of metal ions (Na^+^, K^+^, Fe^2+^, Mg^2+^, Zn^2+^, Co^2+^, Ca^2+^, Mn^2+^, Cu^2+^, Hg^2+^, and Fe^3+^); and 5 mM and 10 mM concentration of inhibitors/activators (ethylene-di-amine tetra acetic acid (EDTA), sodium dodecyl sulfate (SDS), β-mercaptoethanol, phenyl methyl sulfonyl fluoride (PMSF), diisopropyl fluorophosphates (DFP), 1,10 phenanthroline, dithiothreitol (DTT), and Tween 20), which were then tested for fibrin hydrolysis. The activity and stability of the fibrinolytic protease towards organic solvents (10%) like methanol, ethanol, toluene, acetonitrile, chloroform, benzene, hexane, DMSO, 1-propanol, and 2-propanol were also examined. The solution without any treatment served as control.

### 2.13. In Vitro Thrombolytic Activity of the Fibrinolytic Protease Using Blood Clot as Substrate

#### 2.13.1. Blood Clot Preparation

The approval of participants for collection of mammalian blood samples was obtained from the Committee on Institutional Ethical Clearance (IEC), Amity University, Noida, UP, (AUUP/IEC/2018-AUG-03). The signed informed consent was obtained from the participants for the study and all the experiments were performed in accordance with the relevant guidelines and regulations. Venous blood via phlebotomy using vacuum extraction system with sterile needles was drawn from healthy volunteers (20 mL) with O blood group to ensure consistency in results. The sample was further transferred into 20 pre-weighed sterile Eppendorf tubes (1 mL each) free of anti-coagulant, and was incubated at 37 °C for 15–20 min, inducing clot formation. The weight of clot formed was determined (weight = weight of tube with clot – weight of empty tube) using analytical electronic weighing balance. The clots were heated at 80 °C for 45 min to deactivate the endogenous fibrinolytic factors such as plasmin and plasminogen.

#### 2.13.2. Clot Lysis

The efficacy of the purified fibrinolytic protease in clot dissolution was evaluated by its in vitro thrombolytic activity analysis in Eppendorf tubes. The concentration of fibrinolytic protease was fixed (100 Units) and the effect of incubation time (1, 2, 3, and 4 h) on clot lysis was assessed. With the fixed incubation period (4 h), the efficacy of fibrinolytic protease was studied in comparison to a laboratory protease and thrombolytic agent. A non-fibrinolytic laboratory protease by *Bacillus megaterium* B69 [49] and commercial streptokinase/myokinase from Biocon, India (1,500,000 IU) were used as per the protocol for the blood clot dissolution assay. The effect of different concentrations (25, 50, 75, and 100 Units) of the two different proteases and streptokinase with blood clot was observed. The samples were observed for percentage weight dissolution of blood clots within 4 h of incubation at 37 °C. The tubes were centrifuged at 30,000 rpm for 10 min and the Eppendorf tubes, along with pellet (blood clot) were weighed.

## 3. Results and Discussion

### 3.1. Isolation and Screening of Bacterial Strain Producing Fibrinolytic Protease

One hundred and thirty bacterial strains out of 400 isolates were screened for protease production on skimmed milk agar plates. *Bacillus* RSA1, RSA3, RSE163, RSA7, RSB18, RSB12, RSC117, RSA5, and RSB17 showed maximum hydrolytic zone on the skimmed milk agar plates, which were further screened for fibrinolytic activity on fibrin plates. The milk hydrolyzing capability was used for the primary screening of protease producing *Bacillus* strains [44,52].

### 3.2. Fibrinolytic Activity of the Screened Bacterial Strains Using Fibrin Plate

**Figure 1:**
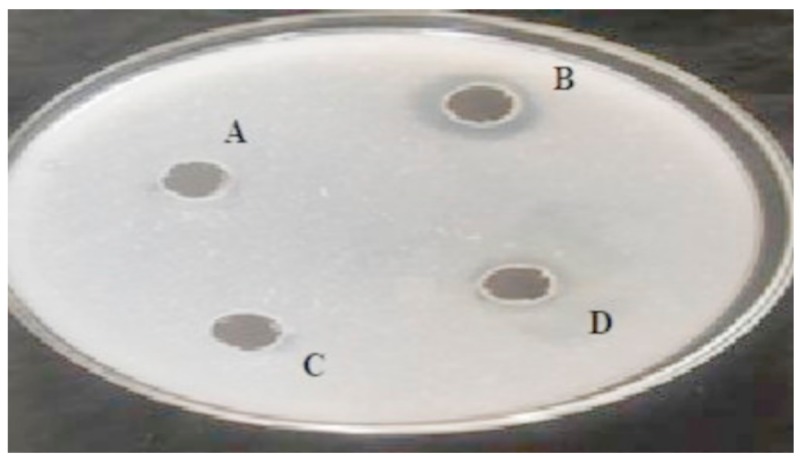
Expression of fibrinolytic protease on fibrin plate by bacterial strains: A: Control; B: RSA1 Strain; C: RSA3 Strain, and D: RSE163 Strain.

Fibrin plate assay revealed three strains (RSA1, RSA3, RSE163) showing high fibrinolytic activity (Figure 1) with a clear transparent region of fibrin hydrolysis. The RSA1 strain exhibited a bigger zone of hydrolysis than RSA3 and RSA163. Thus, the significant inhibition zone demonstrating the enzymatic activity obtained by fibrin plate assay reveals RSA1 strain as a potent source for the production of fibrinolytic protease. Similar significant results were obtained for fibrinolysis using fibrin plate assay performed by Kang et al. and Liu et al. [9,14].

### 3.3. Identification and Phylogenetic Analysis of the Strain

**Figure 2:**
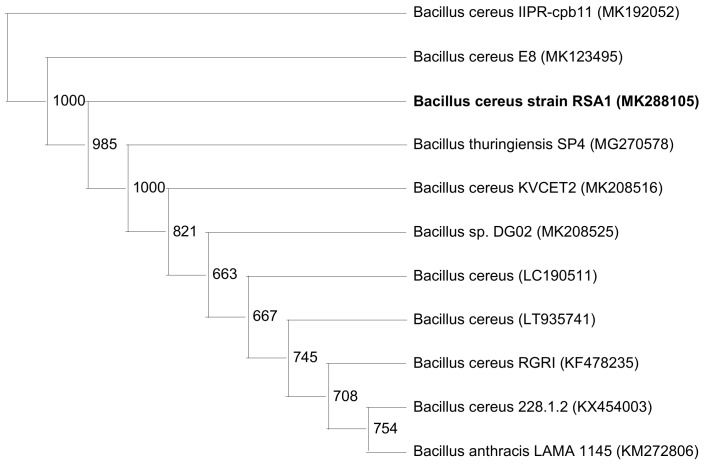
Neighbor-joining phylogenetic analysis showing the relationship of *Bacillus cereus* RSA1 (MK288105) with other *Bacillus* strains. 16S rRNA gene sequences of *B. cereus* RSA1 and other strains, mainly 10 sub-clusters of *Bacillus* sensu stricto group were used for analysis. The bootstrap values are provided at nodes.

The strain RSA1 was observed as gram positive, rod shaped endospore forming *Bacillus* strain. Biochemical characterization revealed no indole production, methyl red, or oxidase activity, while positive results were obtained for the voges–proskauer and catalase test. It utilized citrate, exhibited gelatinase and catalase activities, along with nitrate reduction, and did not show starch utilization or oxidase activity but utilized sugars with gas production [30,43]. The selected strain was further subjected to molecular characterization and was identified as *Bacillus cereus* RSA1 (NCBI Accession number MK288105). The phylogenetic analysis using neighbor-joining methodology provided an in-depth evolutionary relationship of *B. cereus* RSA1 with some inter-related *B. cereus* strains (MK192052, MK123495, MK208516, LC190511, LT935741, KF478235, KX454003) and other species such as *B. thuringiensis* (MG270578), *Bacillus* sp. (MK208525), and *B. anthracis* (KM272806). The obtained phylogenetic tree revealed a distinct phylogenetic position and close association of the newly identified *B. cereus* RSA1 strain within the genus (Figure 2). The high efficiency of the neighbor-joining method in achieving the accurate phylogenetic tree topology is proven in already published research reports as well [50].

### 3.4. Fibrinolytic Activity of Protease and Statistical Optimization of Media

#### 3.4.1. Elucidation of Significant Variables

The Plackett–Burman Design was used to evaluate the most significant variables for enhanced production of fibrinolytic protease from a number of carbon sources (glucose and sucrose), nitrogen sources (peptone, casein, and yeast extract), inorganic salts (K_2_HPO_4_, MgSO_4_), and cultivation parameters (rpm, time of incubation, inoculums size, and initial pH) [30,44,45,46]. The results including the actual level of variables with symbol codes (as mentioned in Section 2.8) are displayed in Table 1 and Table 2. The E(xi) coefficient indicates that nine variables significantly influenced the production of enzyme (peptone, casein, glucose, yeast extract, K_2_HPO_4_, MgSO_4_, incubation time, rpm, and initial pH). Four significant variables (peptone, casein, glucose, and yeast extract) earmarked by Plackett–Burman design to have maximum influence on the fibrinolytic protease production were optimized using CCD, keeping the other parameters fixed.

The F value of the model is 12,455, which indicates it to be significant (Table 3). The predicted *R*^2^ value of the model is 0.9863 and the adjusted *R*^2^ is 0.999, which indicates that the two are in sync with each other. The adequate precision value is 391 (A ratio >4 is required). This indicates that the model is well fitted. 

#### 3.4.2. Optimization of Variables by Response Surface Methodology

Experimental design with predicted response for protease production and maximum fibrinolytic activity (30.75 U/mL) is displayed in Table 4 and Table 5. Table 6 provides the *p*-value of the model, which is <0.05, indicating that the protease activity could be well explained using this model. 

ANOVA results indicate that the model is significant, with a 2.89 F value (Table 6). The variables A, B, and D were significant for the enzyme production. The model can explain 86% variation (*R*^2^ value was 0.8650) in the data. The lack of fit value was 0.71, indicating its non-significance. The adequate precision value was 7.378, showing an adequate signal for the model. The data obtained from the model follow second-order polynomial equation (wherein A is peptone, B is casein, C is yeast extract, D is glucose, and E is sucrose) is as follows Equations (3):

Enzyme production = + 25.86 + 2.67*A + 1.32*B + 2.533E − 003*C + 1.33*D(3)

**Figure 3:**
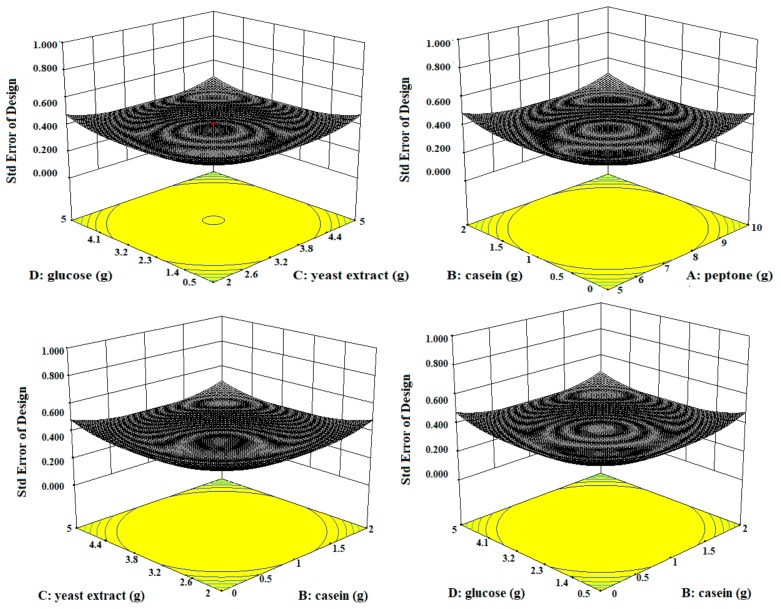

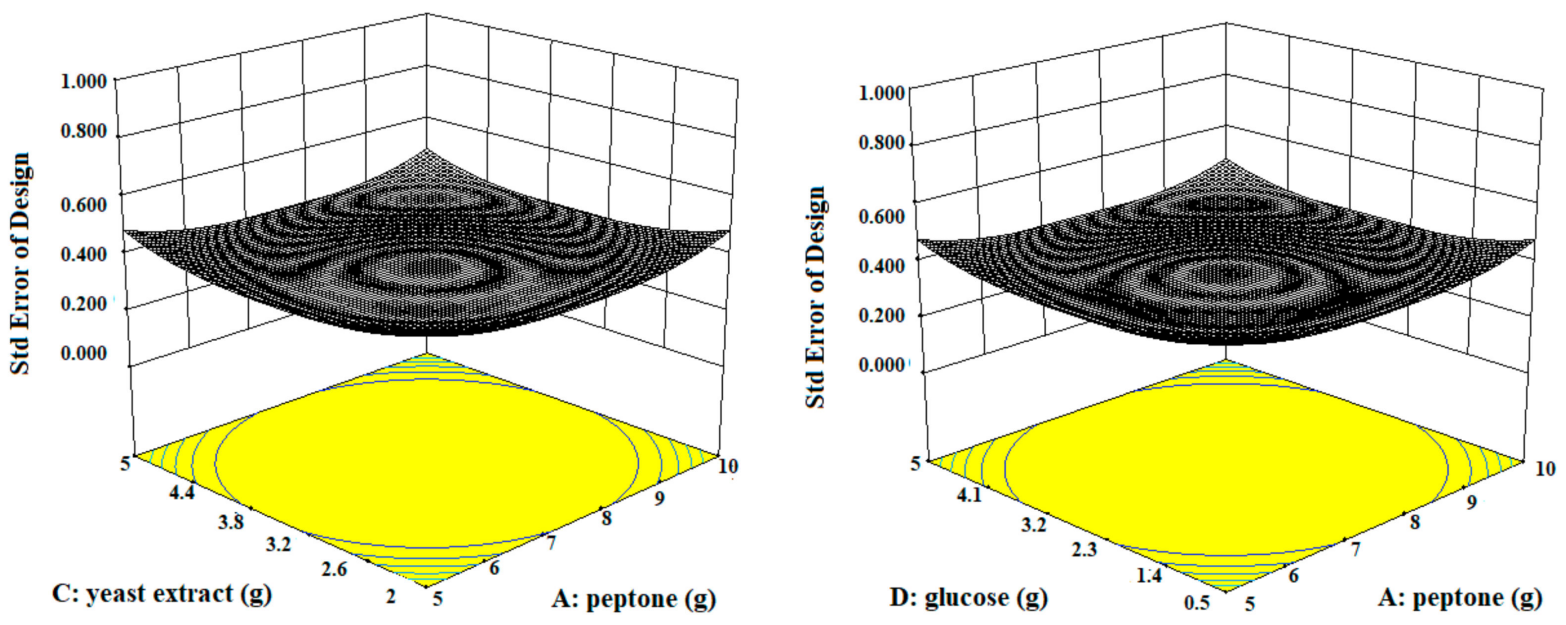
The three-dimensional (3D) contour plots of fibrinolytic protease activity, showing interactions among peptone, casein, yeast extract, and glucose, keeping the other parameters fixed.

Three-dimensional contour plots expedited the recognition of optimal levels of significant variables for fibrinolytic protease production. The 3D plots revealed that the independent variables peptone (10 g/L), yeast extract (5 g/L), and glucose (0.5 g/L) significantly increased the production of fibrinolytic protease (Figure 3).

The impact of all of the involved variables, along with optimization, requires more resources and time. These limitations were overcome by optimizing the influential factors with a reliable statistical tool, such as response surface methodology. Literature has proved the reliability of such tools and statistical optimization has led to about 4-fold and 2-fold increase in the production of enzymes by *Bacillus cereus* IND1 (3699  U/mL) and *Bacillus* sp. (577 U/mL), respectively [30,44].

#### 3.4.3. Validation of the Design Model

A 2-fold increase in the production of fibrinolytic protease was observed using a statistical optimization tool. The obtained fibrinolytic protease was 30.75 U/mL, which was double in production than the initial medium without optimization (15 U/mL). The results from the response surface model were confirmed by conducting the experiments under conditions predicted by the model. The designed model was validated as the predicted response for fibrinolytic protease production and was 32.8 U/mL, which was close to the experimental value (30.75 U/mL). Literature recommends the use of Plackett–Burman design and the CCD model, wherein, researchers have obtained the predicted response 449 U/mL and experimental value 577 U/mL, thus validating the use of model [44]. Other comparative studies also support the use of statistically optimized medium for the production of fibrinolytic enzyme [30].

### 3.5. Purification of Fibrinolytic Protease

**Figure 4:**
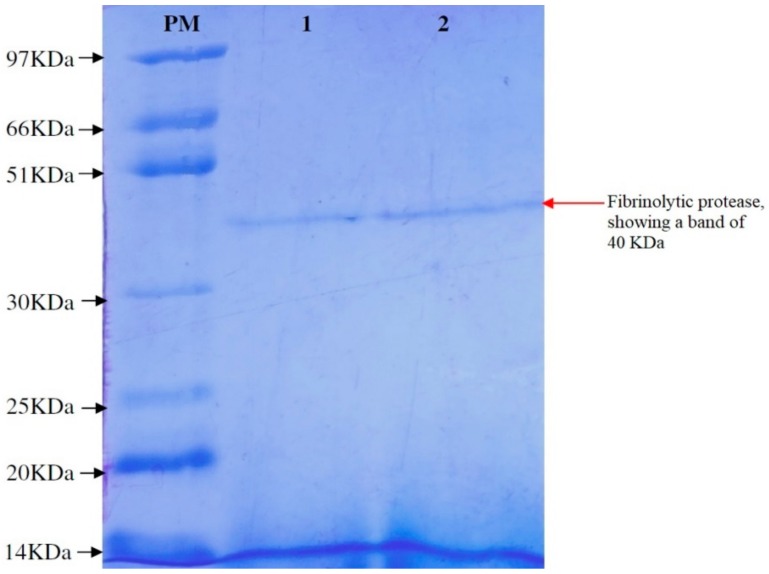
SDS-PAGE of purified fibrinolytic protease. (**PM**) Protein marker; (**Lane 1**) 7.5 µL dialyzed fibrinolytic protease; (**Lane 2**) 15 µL dialyzed fibrinolytic protease.

*Bacillus cereus* RSA1 produced fibrinolytic enzyme underwent chilled ethanol precipitation, wherein we obtained 35.39% recovery with 1.28-fold purification of fibrinolytic enzyme. The enzyme was further purified on a Sephadex G-75 column and the obtained fractions were 2.32-fold enriched with fibrinolytic enzyme. The fibrinolytic enzyme was better purified with 33.11% recovery in comparison to crude cell extract (Table 7). The SDS-PAGE analysis revealed the molecular weight of protein as 40 KDa (Figure 4). Numerous fibrinolytic proteases of *Bacillus* sp. origin have been studied and are reported in literature. Some of these fibrinolytic enzymes exhibit apparently similar molecular masses of 29.5 and 36.12 KDa, and were obtained from *Bacillus cereus* IND1 and *Bacillus licheniformis* UV-9, respectively [30,53]. 

### 3.6. MALDI–TOF MS Analysis

**Figure 5:**
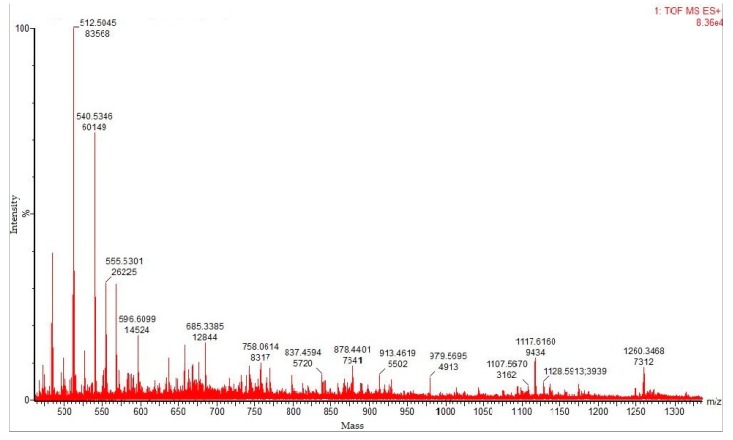
MALDI–TOF mass spectra of purified fibrinolytic protease. The molecular masses of discrete isoforms are designated above the major spectrum peaks.

Matrix-assisted laser desorption ionization–time of flight (MALDI–TOF) mass spectrometric analysis of the tryptic-digested protease resulted in a spectrum of peaks, which provides ration *m*/*z* of peptide fragments, and the purified fibrinolytic protease was found to be a blend of several isoforms (Figure 5). Table 8 provides the details about the *m*/*z* peptide matching data analysis. The MASCOT search revealed that the fibrinolytic protease held 56% similarity with Subtilisin NAT OS = *Bacillus subtilis* subsp. natto OX = 86029 GN = aprN PE = 1 SV = 1, for a sequence score of 381 amino acids (M_r_ 39,483 Da). The matching of 27 values and top score of 189 with Subtilisin NAT OS = *Bacillus subtilis* subsp. natto OX = 86029 GN = aprN PE = 1 SV = 1 (SUBN_BACNA) and Subtilisin E OS = *Bacillus subtilis* (strain 168) OX = 224308 GN = aprE PE = 1 SV = 3 (SUBT_BACSU) was obtained. Further, the NCBI protein blast for sequence homology search conveyed 100% similarity with MULTISPECIES: Subtilisin AprE (*Bacillus*) with reference sequence WP_014479360.1. Subsequently, 99.74% identity, 100% query cover, and max/total score of 766 were observed with nattokinase (*Bacillus subtilis*, AUR45012.1 and AEV91244.1), subtilisin (*Bacillus subtilis*, QDR50097.1), and subtilisin AprE [*Bacillus subtilis*, WP_088272268.1]. The Prosite search revealed that the fibrinolytic protease belong to serine protease (subtilase family), showing three active sites viz. VAVIDSGIdssH (aspartic acid active site, position: 134–145), HGThVAGtIAA (histidine active site, position: 170–180), and GTSmAtPhVAG (serine active site, position: 325–335). The deduced analysis confirms that the fibrinolytic protease exhibit no similarity with any *Bacillus cereus* protein, suggesting that it may be a new and novel fibrinolytic protein. The peptide sequences of fibrinolytic protease from *Bacillus cereus* RSA1 are mentioned underneath, wherein the bold peptides represent the matched ones.

**MRSKKLWISLLFALTLIFTMAFSNMSAQAAGKSSTEKKYIVGFKQTMSAMSSAKKKDVISEKGGKVQKQFKYVNAAAATLDEKAVKE**LKK**DPSVAYVEEDHIAHEYAQSVPYGISQIK**APALHSQGYTGSNVK**VAVIDSGIDSSHPDLNVR**GGASFVPSETNPYQDGSSHGTHVAGTIAALNNSIGVLGVAPSASLYAVKVLDSTGSGQYSWIINGIEWAISNNMDVINMSLGGPTGSTALKTVVDK**AVSSGIVVAAAAGNEGSSGSTSTVGYPAKYPSTIAVGAVNSSNQR**ASFSSVGSELDVMAPGVSIQSTLPGGTYGAYNGTSMATPHVAGAAALILSK**HPTWTNAQVRDRLESTATYLGNSFYYGKGLINVQAAAQ**

Saxena and Singh used MALDI–TOF analysis for the analyses of fibrinolytic protease from *Bacillus cereus* B80, which was found to exhibit 70–93% similarity with zinc metalloprotease specifically from *Bacillus cereus* group [54]. The proteomics study of extracellular fibrinolytic proteases obtained from fermented food (Indonesian) from *Bacillus pumilus* 2.g and *Bacillus licheniformis* RO3 was also accomplished using MALDI–TOF analysis, and found it identical to flagellin of *Bacillus licheniformis* DSM 13. [55].

### 3.7. Characterization of Purified Fibrinolytic Protease

#### 3.7.1. Optimization of Temperature and pH

**Figure 6:**
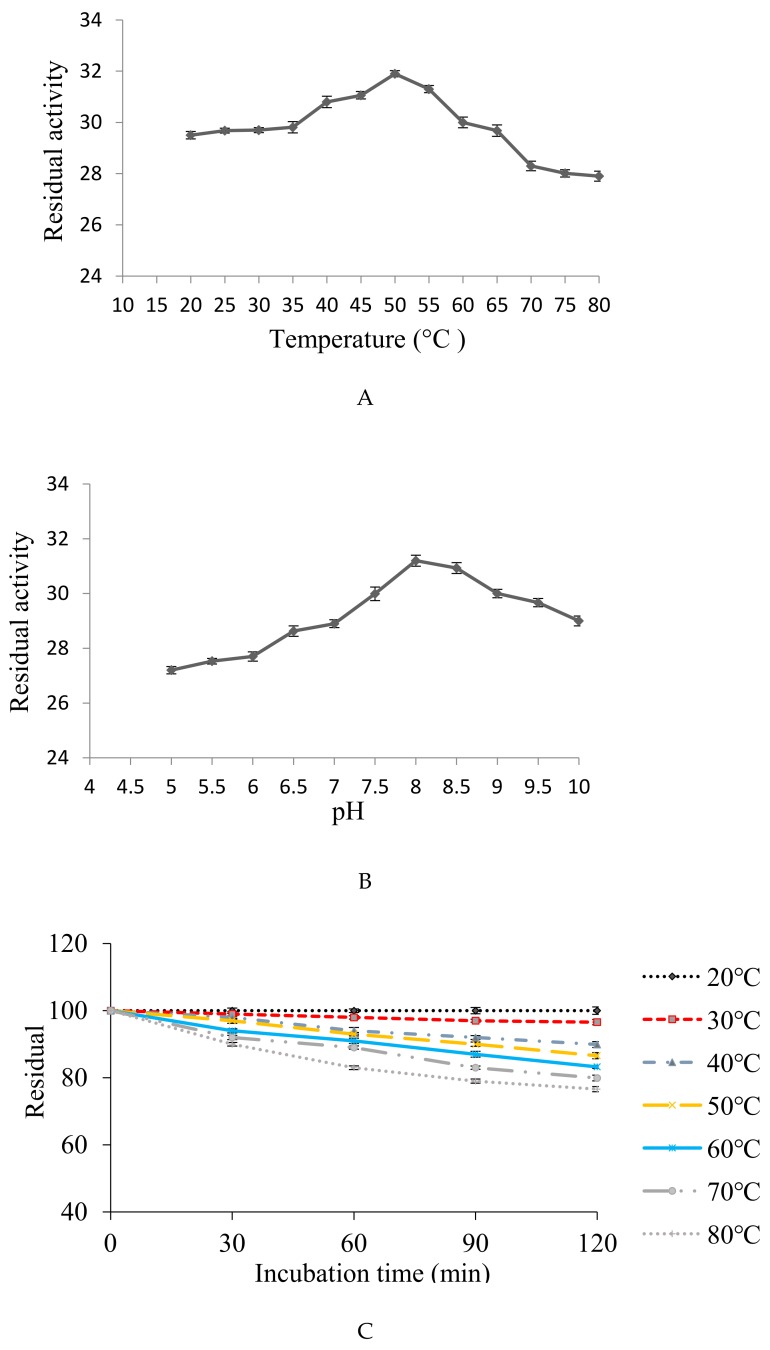

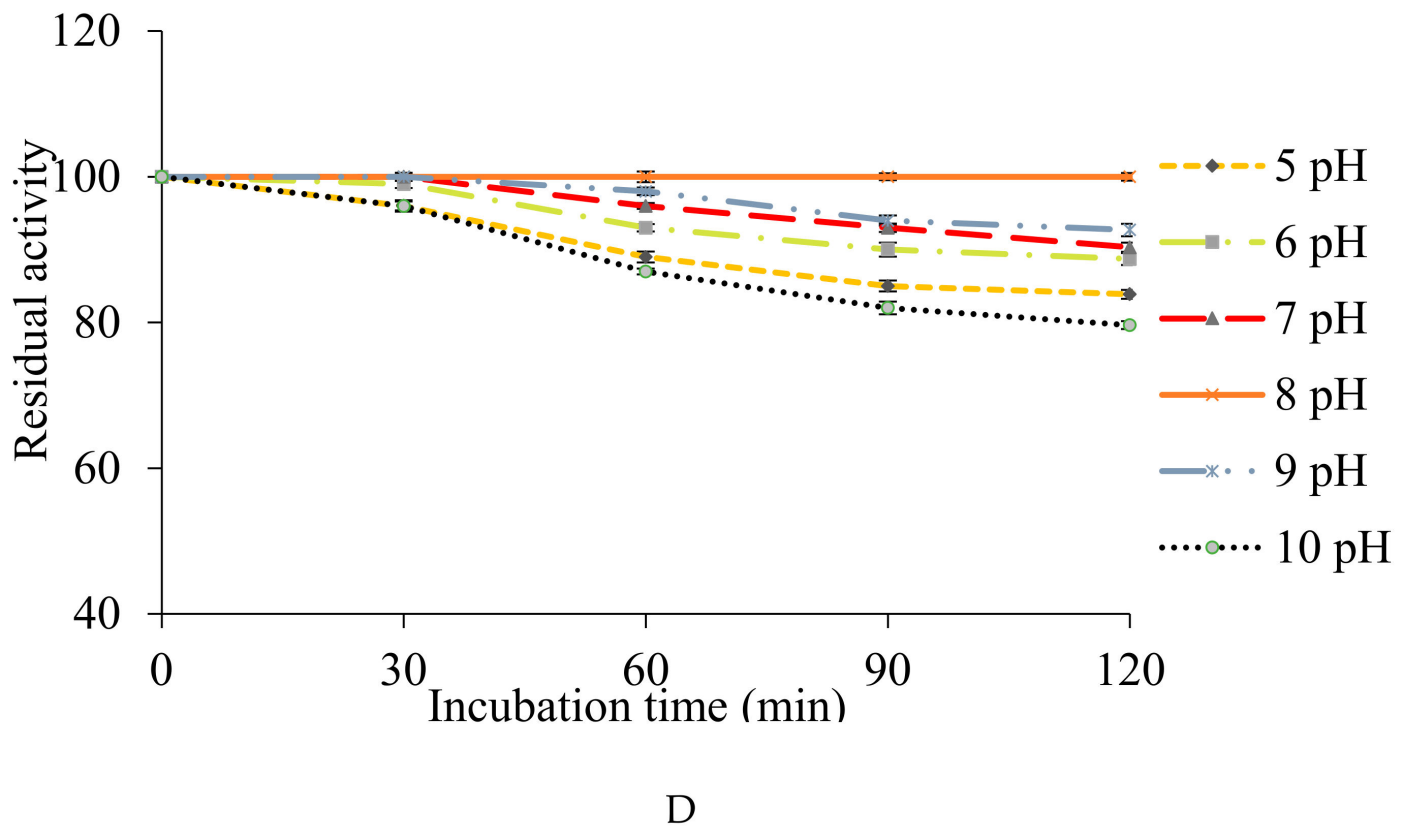
(**A**,**B**) Optimization of temperature and pH, with maximum fibrinolytic protease production at 50 °C and pH 8. (**C**,**D**) Stability of enzyme within a wide range of temperature (20–80 °C) and pH (5–10).

The protease showed maximum fibrinolytic activity at temperature 50 °C. A gradual decrease in activity was observed up to 80 °C with relative activity of 94.04, 93.04, 88.71, 87.80, and 87.46% at 60, 65, 70, 75, and 80 °C, respectively (Figure 6A). The optimal temperature of the fibrinolytic enzyme is similar to the fibrinolytic enzymes obtained from *Bacillus subtilis* KCK-7 [56] and *Bacillus amyloliquefaciens* DC-4 [57], with maximum activity at 60 and 48 °C, correspondingly. 

The protease exhibited significantly commendable fibrinolytic activity within pH range 7–10, with maximum activity at pH 8 (Figure 6B). Similar results were reported for fibrinolytic enzyme produced by *Serratia* sp. KG-2-1 [33], *B. subtilis* HQS-3 [58], and *Pseudoalteromonas* sp., IND11 [59]. Scientific reports indicate that most proteases are active in neutral to alkaline conditions, from pH 7.0 to 9.5 [60], and a few of extreme alkalophilic optima at pH 12–13 [61].

#### 3.7.2. Stability of Fibrinolytic Protease at Different Temperatures and pH Ranges

The obtained fibrinolytic protease was thermostable within a wide temperature range (20–80 °C). Maximum stability (100%) was observed at 20 °C and retained 76.59% of its activity up to 80 °C after 2 h of incubation (Figure 6C). The obtained fibrinolytic enzyme has more stability than those reported earlier, which showed thermo-stability below 40 °C [62] and up to 52 °C [63].

The protease was found stable with 100% of activity at pH 8 after 2 h of incubation. The enzyme exhibited broad pH stability, ranging from pH 5–10 and could retain more than 79.64% of its original activity when incubated for 2 h (Figure 6D). An alkaline serine protease CFR15 produced by *Bacillus amyloliquefaciens* MCC2606 was found stable at pH range 7–10.5 [64]. A broad spectrum pH stability profile (3–11) retaining >90% was reported for fibrinolytic protease produced by *Cordyceps militaris* [41]. A similar pH stability profile (4–11) was reported for fibrinolytic protease with more than 70% of its activity by *Neurospora sitophila* [63].

#### 3.7.3. Kinetic Studies

**Figure 7:**
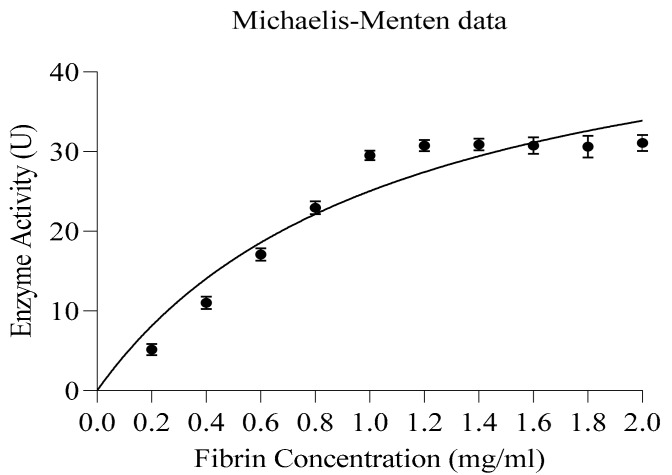
Enzyme kinetics—non-linear regression: Michaelis–Menton plot for kinetic studies.

The reaction kinetics of obtained fibrinolytic protease was determined from a Michaelis–Menten non-linear regression curve (Figure 7) under optimum conditions (temperature and pH). GraphPad Prism 8.Ink was used for non-linear regression analysis. The calculated K_m_ value of the enzyme was 1.093 mg/mL; however, the maximum reaction velocity (V_max_) of the enzyme was 52.39 µg/mL/min protein. The fibrinolytic protease (SFE1) from *Streptomyces* sp. XZNUM 00,004 also showed 0.96 mg/mL K_m_ and 181.8 U/mL V_max_ values [65]. The K_m_ and V_max_ values of the purified protease enzyme from *C. guilliermondii* for fibrin were found to be 5 mg and 66.67 μ/mL/min, respectively [66]. The values of K_m_ and V_max_ were 0.18 mM and 53.5 U/mL, correspondingly, for the enzyme obtained from *Pleurotus eryngii* [67].

#### 3.7.4. Effect of Inhibitors/Activators, Metal Ions, and Organic Solvents on Enzyme Activity 

**Figure 8:**
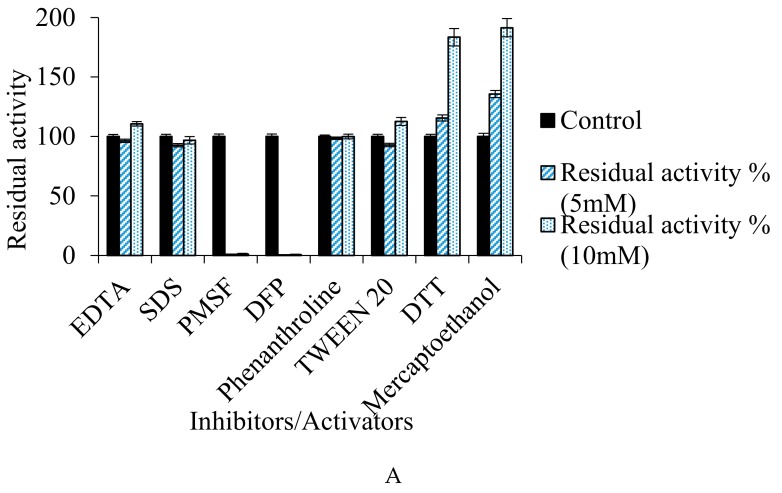

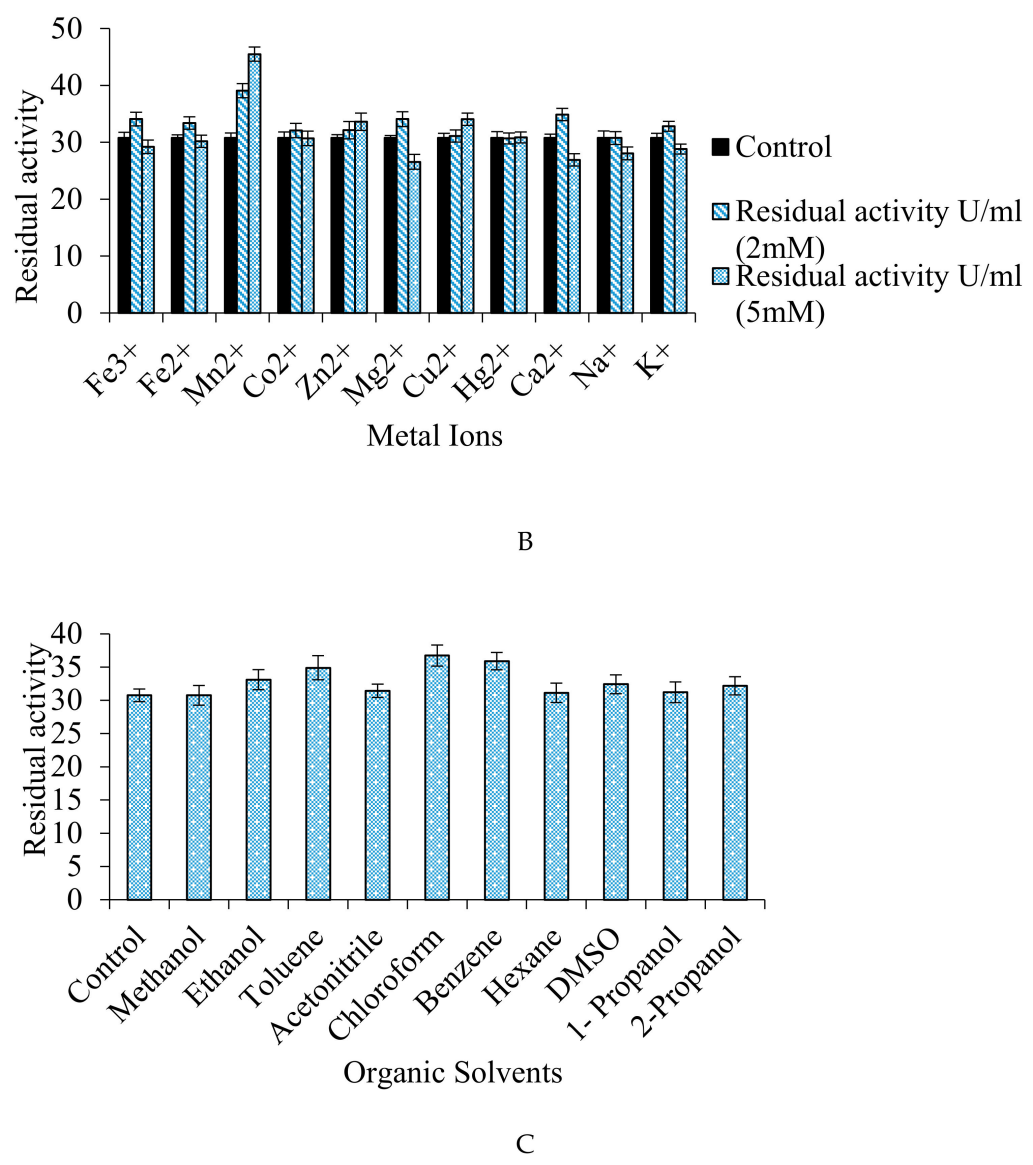
(**A**–**C**) Effect of inhibitors/activators (5 mM and 10 mM), metal ions (2 mM and 5 mM), and organic solvents (10%) on the fibrinolytic activity of the protease.

It was observed that the enzyme retained its stability and activity with SDS (96.77%) and Tween 20 (112.59%). Also, the enzyme activity was neither reduced by 1,10 phenanthroline (98.56%) nor EDTA (110.59%), showing that the protease does not belong to metallo-category (Figure 8A). The fibrinolytic enzyme obtained from *Cordyceps militaris* was neither inhibited by EDTA nor by PMSF, but was completely inhibited by aprotinin and soybean trypsin inhibitor [41]. On the other hand, enzyme from *Bacillus subtilis* ICTF-1 [65] and *Mucor subtillissimus* UCP 1262 [68] were inhibited by PMSF (1 mM), while the enzyme from *B. pumilus* 2.g, was totally inhibited by EDTA (1 mM) [69]. 

However, complete loss in activity of fibrinolytic protease from *Bacillus cereus* RSA1 was observed with PMSF (0.99%) and DFP (0.39%), and there was ≈2-fold increase in the activity with β-mercaptoethanol (191.41%) and DTT (183.42%), which indicates it to be a thiol-dependent serine fibrinolytic protease (Figure 8A). The present study is supported by the protease produced by *Bacillus cereus* PMW8 [70]. Thiol-dependent protease was also obtained from *Exiguobacterium* sp. SKPB5 (MTCC 7803) [71]. Also, *Bacillus mojavensis* produced a thiol-dependent protease with 2-fold increase in activity in the presence of DTT and β-mercaptoethanol [72].

The effect of various monovalent, divalent, and trivalent metal ions was also studied and it was revealed that the Mn^2+^ (45.49 U/mL), Zn^2+^ (33.61 U/mL), and Cu^2+^ (34.05 U/mL) ions enhanced the fibrinolytic activity of the protease (Figure 8B). The effect of organic solvents, which were 10% mixture in water, such as methanol (30.75 U/mL), ethanol (33.10 U/mL), toluene (34.91 U/mL), acetonitrile (31.43 U/mL), chloroform (36.73 U/mL), benzene (35.90 U/mL), hexane (31.12 U/mL), DMSO (32.41 U/mL), 1-propanol (31.20 U/mL), and 2-propanol (32.18 U/mL) on protease when studied, revealed that the protease retained 100% of its activity with all solvents, and thus may have wide industrial applications (Figure 8C). The strains like *B. lehensis* [73], *Bacillus* sp. EMB9 [74], and *B. circulans* M34 [75] produced organic solvent solution-tolerant proteases. Fibrinolytic protease by *Bacillus subtilis* K42 [76] and *Serratia* sp. [33] sustained 80–85% of its activity in the presence of organic solvents. Also, acetone, acetic acid, Tween 20, glycerol, isopropanol, DMSO, and SDS stimulated fibrinolytic activity [33].

### 3.8. Fibrinolysis Assay for Blood Clot Dissolution

**Figure 9:**
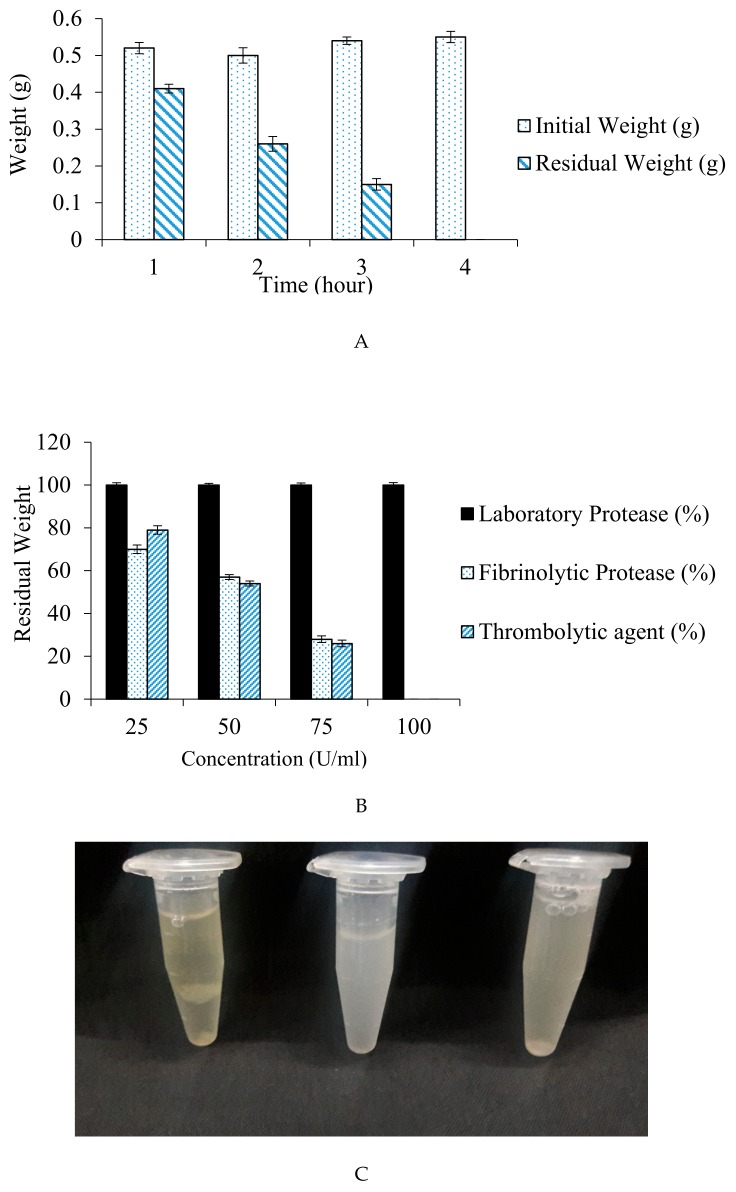
(**A**) Effect of incubation time on the dissolution of blood clot. (**B**) Percentage dissolution of clot in different concentration of fibrinolytic, laboratory and streptokinase. (**C**) Comparative analysis for the efficacy of fibrinolytic protease, a commercialized thrombolytic agent, and laboratory protease in mammalian blood clot dissolution: (1) Laboratory protease: No blood clot dissolution; (2) commercialized streptokinase: Blood clot dissolves completely within 4 h of incubation; (3) fibrinolytic protease: Complete blood clot dissolution occurs within 4 h of incubation.

The increase in the incubation time has led to a significant difference in percentage weight dissolution of the blood clot. Residual weight obtained after incubation period of 1, 2, 3, and 4 h was 0.41, 0.26, 0.15, and 0.00 g, correspondingly. The result (Figure 9A) depicted complete blood clot dissolution within 4 h at 37 °C. 

Also, no blood clot dissolution was observed when a purified protease of our laboratory collection was used for fibrinolytic assay. A significant decrease in clot weight with 25, 50, and 75 U/mL concentration of fibrinolytic protease was observed with 70, 57, and 22% residual clot weight, respectively. Blood clot dissolution was 79, 54, and 26% with commercially available thrombolytic agent (Streptokinase) that had 25, 50, and 75 U/mL concentration, respectively. The clot completely disappeared (100% dissolution) using 100 Units of fibrinolytic protease and streptokinase at 37 °C within 4 h. The result is displayed in Figure 9B.

Compared to current alternatives, the degradation of halo clots with our fibrinolytic protease is very precise and highly efficacious. Lu and Chen reported thrombolytic effects of fibrinolytic protease (0.2 and 0.5 μg) derived from *Schizophyllum commune* using mammalian (rat) blood, wherein they observed complete blood clot dissolution after 8 h of incubation [77]. Complete blood clot dissolution was observed within 5 h in a dog model when orally administered with nattokinase capsules [78]. Also, blood clot dissolution was obtained in 2 h using cell-free extracts of *B. subtilis* G8, whereas *E. coli* ATCC 8739 resulted in no blood clot size reduction even after 6 h of incubation [79].

The results indicate that thiol-dependent fibrinolytic protease isolated by *Bacillus cereus* RSA1 has tremendous possibilities towards pharmacological application as a prodigious thrombolytic agent. The embellishment of fibrinolytic activity due to the stability towards a wide range of temperatures, pH levels, metal ions, inhibitors, surfactants, and solvents aids in overcoming the numerous confines of the prevalent conventional fibrinolytic approaches. The present thiol-dependent fibrinolytic protease completely dissolves fibrin clots in comparison with a marketed drug, which indicates its potential use as a perceptible agent for oral fibrinolytic therapy and/or in wound healing processes. In summation, therapeutic application of protease can be acclimatized to the health services as prudence for the management and treatment of cardio-vascular disorders.

## Figures and Tables

**Table 1:** Set of 13 run experiments with the 11 selected variables created using the Design Expert 10.0.8.0.

Run	Peptone (g)	Yeast Extract (g)	Casien (g)	Glucose (g)	Sucrose (g)	K_2_HPO_4_ (g)	MgSO_4_ (g)	Speed (rpm)	pH	Time (h)	Inoculum (%)	Response (U/mL)
1	5	5	2	0.5	5	4	1	100	7	24	5	16.5
2	7.5	3.5	1	2.75	2.5	2.5	0.75	140	8	48	3	17.9
3	5	2	0	0.5	0	1	0.5	100	7	24	1	6.5
4	10	2	0	0.5	5	1	1	180	7	72	5	13.2
5	10	2	2	5	5	1	0.5	100	9	24	5	24.7
6	10	5	0	5	5	4	0.5	100	7	72	1	23.0
7	5	2	0	5	0	4	1	100	9	72	5	17.6
8	5	5	0	5	5	1	1	180	9	24	1	14.6
9	5	5	2	5	0	1	0.5	180	7	72	5	16.9
10	10	5	0	0.5	0	4	0.5	180	9	24	5	17.8
11	5	2	2	0.5	5	4	0.5	180	9	72	1	10.6
12	10	2	2	5	0	4	1	180	7	24	1	26.1
13	10	5	2	0.5	0	1	1	100	9	72	1	28.6

**Table 2:** Plackett–Burman Design indicating the ranking of variables which significantly influence the enzyme production.

Variable	Component	Mi+	Mi−	E(xi)	Absolute E(xi)	Ranking
A	Peptone	133.4	82.7	8.45	8.45	1
B	Yeast extract	117.4	98.7	3.11	3.11	4
C	Casein	123.4	92.7	5.116	5.116	2
D	Glucose	122.9	93.2	4.95	4.95	3
E	Sucrose	102.6	113.5	−1.8166	1.8166	9
F	K_2_HPO_4_	111.6	104.5	1.1833	1.833	8
G	MgSO_4_	116.6	99.5	2.85	2.85	6
H	RPM	116.9	99.2	2.95	2.95	5
J	pH	113.9	102.2	1.95	1.95	7
K	Incubation time	109.9	106.2	0.616	0.616	10
L	Inoculum size	106.7	109.4	−0.45	0.45	11

**Table 3:** ANOVA for Placket-Burman.

Source	Sum of Squares	Degree of Freedom	Mean Square	F Value	*p*-Value Prob > F	
Model	473.10	11	43.01	12,455.28	0.0070	Significant
Peptone	213.72	1	213.72	61,893.00	0.0026	
Yeast extract	29.42	1	29.42	8519.68	0.0069	
Casein	79.19	1	79.19	22,932.00	0.0042	
Glucose	73.27	1	73.27	21,218.08	0.0044	
Sucrose	9.83	1	9.83	2847.52	0.0119	
K_2_HPO_4_	4.31	1	4.31	1248.52	0.0180	
MgSO_4_	24.16	1	24.16	6997.12	0.0076	
RPM	26.29	1	26.29	7613.32	0.0073	
pH	11.21	1	11.21	3245.32	0.0112	
Incubation time	1.12	1	1.12	325.00	0.0353	
Inoculum size	0.58	1	0.58	168.48	0.0489	
Residual	0.00345	1	0.00345			
Cor total	473.10	12				

**Table 4:** Central composite design (CCD) summary.

Variable	Name	Units	Type	Subtype	Minimum	Maximum	−1 Actual	+1 Actual	Mean	Std. Dev.
A	Peptone	G	Numeric	Continuous	2.5	12.5	5	10	7.5	2.27429
B	Casein	G	Numeric	Continuous	−1	3	0	2	1	0.909718
C	Yeast extract	G	Numeric	Continuous	0.5	6.5	2	5	3.5	1.36458
D	Glucose	G	Numeric	Continuous	−1.75	7.25	0.5	5	2.75	2.04686

**Table 5:** Central composite design (CCD) response.

Response Name	Units	Obs	Analysis	Minimum	Maximum	Mean	Std. Dev.	Ratio	Trans	Model
Fibrinolytic protease activity	U/mL	30	Polynomial	15.1193	30.7523	22.4807	4.25754	2.03398	None	2FI

**Table 6:** ANOVA for response surface quadratic model—CCD.

Source	Sum of Squares	Degree of Freedom	Mean Square	F Value	*p*-Value Prob > F	
Model	317.12	10	31.71	2.89	0.0224	Significant
A—Peptone	1.40	1	1.40	0.13	0.7249	
B—Casein	5.25	1	5.25	0.48	0.4974	
C—Yeast extract	2.67	1	2.67	0.24	0.6277	
D—Glucose	116.03	1	116.03	10.57	0.0042	
AB	8.73	1	8.73	0.80	0.3837	
AC	9.17	1	9.17	0.84	0.3723	
AD	65.48	1	65.48	5.97	0.0245	
BC	38.69	1	38.69	3.52	0.0759	
BD	12.28	1	12.28	1.12	0.3034	
CD	57.43	1	57.43	5.23	0.0338	
Residual	208.55	19	10.98			
Lack of fit	138.92	14	9.92	0.71	0.7178	Not Significant
Pure error	69.64	5	13.93			
Cor total	525.67	29				

Std. Dev. 3.31; R-Squared 0.6033; Adj R-Squared 0.3945; Pred R-Squared 0.0102; Adeq Precision 7.378.

**Table 7:** Purification parameters of fibrinolytic enzyme from *Bacillus cereus* RSA1.

Purification Steps	Volume (mL)	Total Activity (U)	Total Protein (mg)	Specific Activity (U/mg)	Yield or Recovery (%)	Fold Purification
Crude	100	190	15.4	12.337 ± 0.32	100	1
EtOH-treated	100	86	5.45	15.779 ± 0.45	35.39	1.28
Sephadex-75	100	146	5.1	28.627 ± 0.33	33.11	2.32

**Table 8:** The peptide matching for *m*/*z* data of serine fibrinolytic protease from *Bacillus cereus* RSA1.

Start	End	Observed	Mr(expt)	Mr(calc)	ppm	M	Peptide
1	5	649.3323	648.3250	648.3741	−75.7	2	-.MRSKK.L
5	32	3073.6745	3072.6672	3072.6544	4.17	1	K.KLWISLLFALTLIFTMAFSNMSAQAAGK.S
6	32	2945.5436	2944.5363	2944.5594	−7.85	0	K.LWISLLFALTLIFTMAFSNMSAQAAGK.S
33	44	1386.6593	1385.6520	1385.7554	−74.6	2	K.SSTEKKYIVGFK.Q
38	44	854.5358	853.5285	853.5062	26.2	1	K.KYIVGFK.Q
39	54	1748.8624	1747.8551	1747.8637	−4.90	1	K.YIVGFKQTMSAMSSAK.K
45	54	1041.4160	1040.4087	1040.4630	−52.2	0	K.QTMSAMSSAK.K
45	55	1169.5683	1168.5610	1168.5580	2.59	1	K.QTMSAMSSAKK.K
55	62	946.5210	945.5137	945.5495	−37.8	2	K.KKDVISEK.G
56	65	1060.5299	1059.5226	1059.5924	−65.9	2	K.KDVISEKGGK.V
57	65	932.5373	931.5300	931.4975	35.0	1	K.DVISEKGGK.V
57	68	1287.7175	1286.7102	1286.7194	−7.14	2	K.DVISEKGGKVQK.Q
63	71	1019.6070	1018.5997	1018.5924	7.23	2	K.GGKVQKQFK.Y
66	71	777.4271	776.4198	776.4545	−44.6	1	K.VQKQFK.Y
66	83	2024.3001	2023.2928	2023.0738	108	2	K.VQKQFKYVNAAAATLDEK.A
69	86	1967.2133	1966.2060	1966.0523	78.2	2	K.QFKYVNAAAATLDEKAVK.E
72	83	1265.6468	1264.6395	1264.6299	7.61	0	K.YVNAAAATLDEK.A
72	86	1563.7549	1562.7476	1562.8304	−53.0	1	K.YVNAAAATLDEKAVK.E
91	118	3145.4957	3144.4884	3144.5040	−4.95	0	K.DPSVAYVEEDHIAHEYAQSVPYGISQIK.A
134	151	1893.9589	1892.9516	1892.9592	−4.01	0	K.VAVIDSGIDSSHPDLNVR.G
248	276	2595.2804	2594.2731	2594.2824	−3.57	0	K.AVSSGIVVAAAAGNEGSSGSTSTVGYPAK.Y
277	292	1663.7859	1662.7786	1662.8325	−32.4	0	K.YPSTIAVGAVNSSNQR.A
344	353	1209.5433	1208.5360	1208.6051	−57.1	0	K.HPTWTNAQVR.D
344	355	1480.7394	1479.7321	1479.7331	−0.67	1	K.HPTWTNAQVRDR.L
354	371	2084.9103	2083.9030	2083.9851	−39.4	1	R.DRLESTATYLGNSFYYGK.G
356	381	2779.7205	2778.7132	2778.3864	118	1	R.LESTATYLGNSFYYGKGLINVQAAAQ.-
372	381	984.5695	983.5622	983.5400	22.6	0	K.GLINVQAAAQ.-

## References

[1] World Heart Federation The World’s Most Common Cause of Death, Cardiovascular Diseases (CVDs), Global Facts and Figures. https://www.world-heart-federation.org/wp-content/uploads/2017/05/WCC2016_CVDs_infographic.pdf.

[2] Benjamin E.J., Virani S.S., Callaway C.W., Chamberlain A.M., Chang A.R., Cheng S., Chiuve S.E., Cushman M., Delling F.N., Deo R. (2018). Heart disease and stroke statistics-2018 update: A report from the American Heart Association. Circulation.

[3] Riddel J.P., Aouizerat B.E., Miaskowski C., Lillicrap D.P. (2007). Theories of blood coagulation. J. Pediatr. Oncol. Nurs..

[4] Jeong Y.K., Kim J.H., Gal S.W., Kim J.E., Park S.S., Chung K.T., Kim Y.H., Kim B.W., Joo W.H. (2004). Molecular cloning and characterization of the gene encoding a fibrinolytic enzyme from *Bacillus subtilis* strain A1. World J. Microbiol. Biotechnol..

[5] Mukherjee A.K., Rai S.K., Thakur R., Chattopadhyay P., Kar S.K. (2012). Bafibrinase: A non-toxic, non-hemorrhagic, direct-acting fibrinolytic serine protease from *Bacillus* sp. strain AS-S20-I exhibits in vivo anticoagulant activity and thrombolytic potency. Biochimie.

[6] Mahajan P.M., Nayak S., Lele S.S. (2012). Fibrinolytic enzyme from newly isolated marine *Bacillus subtilis* ICTF-1: Media optimization, purification and characterization. J. Biosci. Bioeng..

[7] Gurewich V. (2016). Thrombolysis: A critical first-line therapy with an unfulfilled potential. Am. J. Med..

[8] Choi J.H., Sapkota K., Park S.E., Kim S., Kim S.J. (2013). Thrombolytic, anticoagulant and antiplatelet activities of codiase, a bifunctional fibrinolytic enzyme from *Codium fragile*. Biochimie.

[9] Kang S.R., Choi J.H., Kim D.W., Park S.E., Sapkota K., Kim S., Kim S.J. (2016). A bifunctional protease from green alga *Ulva pertusa* with anticoagulant properties: Partial purification and characterization. J. Appl. Phycol..

[10] Fenton J.W., Ofosu F.A., Brezniak D.V., Hassouna H.I. (1993). Understanding thrombin and hemostasis. Hematol. Oncol. Clin. N. Am..

[11] Turner N.A., Moake J. (2013). Assembly and activation of alternative complement components on endothelial cell-anchored ultra-large von Willebrand factor links complement and hemostasis-thrombosis. PLoS ONE.

[12] Monroe D.M., Hofman M., Roberts H.R. (2002). Platelets and thrombin generation. Arterioscler. Thromb. Vasc. Biol..

[13] Gabriela C.M., Hajjar K.A. (2005). Molecular mechanisms of fibrinolysis. Br. J. Haematol..

[14] Liu X.L., Zheng X.Q., Qian P.Z., Kopparapu N.K., Deng Y.P., Nonaka M., Harada N. (2014). Purification and characterization of a novel fibrinolytic enzyme from culture supernatant of *Pleurotus ostreatus*. J. Microbiol. Biotechnol..

[15] Bajaj B.K., Singh S., Khullar M., Singh K., Bhardwaj S. (2014). Optimization of fibrinolytic protease production from *Bacillus subtilis* I-2 using agro-residues. Arch. Biol. Technol..

[16] Simkhada J.R., Mander P., Cho S.S., Yoo J.C. (2010). A novel fibrinolytic protease from *Streptomyces sp.* CS684. Process. Biochem..

[17] Kotb E. (2014). The biotechnological potential of fibrinolytic enzymes in the dissolution of endogenous blood thrombi. Biotechnol. Progr..

[18] Nazari J., Davison R., Kaplan K., Fintel D. (1987). Adverse reactions to thrombolytic agents implications for coronary reperfusion following myocardial infarction. Med. Toxicol. Adverse. Drug Exp..

[19] Bonnard T., Law L.S., Tennant Z., Hagemeyer C.E. (2017). Development and validation of a high throughput whole blood thrombolysis plate assay. Sci. Rep..

[20] Bhargavi P.L., Prakasham R.S. (2013). A fibrinolytic, alkaline and thermostable metalloprotease from the newly isolated *Serratia* sp. RSPB11. Int. J. Biol. Macromol..

[21] Lal V. (2017). Fibrinolytic drug therapy in the management of intravascular thrombosis, especially acute myocardial infarction—A review. Asian J. Pharm. Clin. Res..

[22] Bayoudh A., Gharsallah N., Chamkha M., Dhouib A., Ammar S., Nasri M. (2000). Purification and characterization of an alkaline protease from *Pseudomonas aeruginosa* MN1. J. Ind. Microbiol. Biotechnol..

[23] Patel R., Dodia M., Singh S.P. (2005). Extracellular alkaline protease from a newly isolated haloalkaliphilic *Bacillus sp.* production and optimization. Process. Biochem..

[24] Demina N.S., Lysenko S.V. (1991). Micro-organisms synthesizing enzymes with thrombolytic action. Nauchnye. Doki. Vyss. Shkoly Biol. Nauki..

[25] Kotb E. (2013). Activity assessment of microbial fibrinolytic enzymes. Appl. Microbiol. Biotechnol..

[26] Kotb E. (2012). Fibrinolytic bacterial enzymes with thrombolytic activity. Fibrinolytic Bacterial Enzymes with Thrombolytic Activity.

[27] Fayek K.I., EI-Sayed A.T. (1980). Some properties of two purified fibrinolytic enzymes from *Bacillus subtilis* and *B. polymyxa*. J. Basic Microbiol..

[28] Malke H., Ferretti J.J. (1984). Streptokinase cloning expression and excretion by *Escherichia coli*. Proc. Natl. Acad. Sci. USA.

[29] Wang S., Chen H., Liang T., Lin Y. (2009). A novel nattokinase produced by *Pseudomonas sp*. TKU015 using shrimp shells as substrate. Process. Biochem..

[30] Vijayaraghavan P., Vincent P., Gnana S. (2014). Statistical optimization of fibrinolytic enzyme production using agroresidues by *Bacillus cereus* IND1 and its thrombolytic activity in vitro. BioMed Res. Int..

[31] Uesugi Y., Usuki H., Iwabuchi M., Hatanaka T. (2011). Highly potent fibrinolytic serine protease from *Streptomyces*. Enzyme Microb. Technol..

[32] Vijayaraghavan P., Vincent S.G.P., Arasu M.V. (2016). Purification, characterization of a novel fibrinolytic enzyme from *Paenibacillus* sp. IND8, and its in vitro thrombolytic activity. South J. Biol. Sci..

[33] Taneja K., Bajaj B.K., Kumar S., Dilbaghi N. (2017). Production, purification and characterization of fibrinolytic enzyme from *Serratia* sp. KG-2-1 using optimized media. 3 Biotech..

[34] Verma P., Chatterjee S., Keziah M.S., Devi S.C. (2018). Fibrinolytic Protease from Marine *Streptomyces rubiginosus* VITPSS1. Cardiovasc. Hematol. Agents Med. Chem..

[35] EI-Aassar S.A., EI-Badry H.M., Abdel-Fattah A.F. (1990). The biosynthesis of proteases with fibrinolytic activity in immobilized cultures of *Penicillium chrysogenum* H9. Appl. Microbiol. Biotechnol..

[36] Wu B., Wu L., Chen D., Yang Z., Luo M. (2009). Purification and characterization of a novel fibrinolytic protease from *Fusarium* sp. CPCC 480097. J. Ind. Microbiol. Biotechnol..

[37] Ueda M., Kubo T., Miyatake K., Nakamura T. (2007). Purification and characterization of fibrinolytic alkaline protease from *Fusarium* sp. BLB. Appl. Microbiol. Biotechnol..

[38] Batomunkueva B.P., Egorov N.S. (2001). Isolation, purification and resolution of the extracellular proteinase complex of *Aspergillus ochraceus* 513 with fibrinolytic and anticoagulant activities. Microbiology.

[39] Shirasaka N., Naitou M., Okamura K., Fukuta Y., Terashita T., Kusuda M. (2012). Purification and characterization of a fibrinolytic protease from *Aspergillus oryzae* KSK-3. Mycoscience.

[40] Xiao-Lan L., Lian-xiang D., Fu-ping L., Xi-qun Z., Jing X. (2005). Purification and characterization of a novel fibrinolytic enzyme from *Rhizopus chinensis* 12. Appl. Microbiol. Biotechnol..

[41] Liu X., Kopparapu N.K., Li Y., Deng Y., Zheng X. (2016). Biochemical characterization of a novel fibrinolytic enzyme from *Cordyceps militaris*. Int. J. Biol. Macromol..

[42] Nehete P.N., Shah V.D., Kothari R.M. (1985). Profiles of alkaline protease production as a function of composition of the slant, age, transfer and isolate number and physiological state of culture. Biotechnol. Lett..

[43] Narasimhan M.K., Chandrasekaran M., Rajesh M. (2015). Fibrinolytic enzyme production by newly isolated *Bacillus cereus* SRM-001 with enhanced in-vitro blood clot lysis potential 2015. J. Gen. Appl. Microbiol..

[44] Saxena R., Singh R. (2010). Statistical optimization of conditions for protease production from *Bacillus* sp.. Acta Biol. Szeged.

[45] Plackett R.L., Burman J.P. (1946). The design of optimum multifactorial experiments. Biometrika.

[46] Ghanem N.B., Yusef H.H., Mahrouse H.K. (2000). Production of *Aspergillus terreus* xylanase in solid-state cultures: Application of the Plackett-Burman experimental design to evaluate nutritional requirements. Bioresour. Technol..

[47] Astrup T., Mullertz S. (1952). The fibrin plate method for estimating fibrinolytic activity. Arch. Biochem. Biophys..

[48] Moore E., Arnscheidt A., Krüger A., Strömpl C., Mau M. (1999). Simplified protocols for the preparation of genomic DNA from bacterial cultures. Molecular Microbial Ecology Manual.

[49] Saxena R., Singh R. (2014). Contemporaneous production of amylase and protease through CCD response surface methodology by newly isolated *Bacillus megaterium* Strain B69. Enzyme Res..

[50] Saitou N., Nei M. (1987). The neighbor-joining method—A new method for reconstructing phylogenetic trees. Mol. Biol. Evol..

[51] Wang Y.H., Feng J.T., Zhang Q., Zhang X. (2007). Optimization of fermentation condition for antibiotic production by *Xenorhabdus nematophila* with response surface methodology. J. Appl. Microbiol..

[52] Saxena R., Singh R. (2010). Metal ion and pH Stable Protease Production Using Agro-industrial Waste. J. Ecobiotechnol..

[53] Nadeem M., Qazi J.I., Syed Q., Gulsher M. (2013). Purification and characterization of an alkaline protease from *Bacillus licheniformis* UV-9 for detergent formulations. Songklanakarin J. Sci. Technol..

[54] Saxena R., Singh R. (2015). MALDI-TOF MS and CD Spectral Analysis for Identification and Structure Prediction of a Purified, Novel, Organic Solvent Stable, Fibrinolytic Metalloprotease from *Bacillus cereus* B80. BioMed Res. Int..

[55] Afifah D.N., Rustanti N., Anjani G., Syah D., Suhartono M.T. (2017). Proteomics study of extracellular fibrinolytic proteases from *Bacillus licheniformis* RO3 and *Bacillus pumilus* 2.g isolated from Indonesian fermented food. IOP Conf. Ser. Earth Environ. Sci..

[56] Paik H.D., Lee S.K., Heo S., Kim S.Y., Lee H.H., Kwon T.J. (2004). Purification and characterization of the fibrinolytic enzyme produced by *Bacillus subtilis* KCK-7 from Chungkookjang. J. Microbiol. Biotechn..

[57] Peng Y., Huang Q., Zhang R.H., Zhang Y.Z. (2003). Purification and characterization of a fibrinolytic enzyme produced by *Bacillus amyloliquefaciens* DC-4 screened from douchi, a traditional Chinese soybean food. Comp. Biochem. Physiol. B Biochem. Mol. Biol..

[58] Huang S., Pan S., Chen G., Huang S., Zhang Z., Li Y., Liang Z. (2013). Biochemical characteristics of a fibrinolytic enzyme purified from a marine bacterium, *Bacillus subtilis* HQS-3. Int. J. Biol. Macromol..

[59] Vijayaraghavan P., Raj S.R.F., Vincent S.G.P. (2015). Purification and characterization of fibrinolytic enzyme from *Pseudoalteromonas* sp., IND11 and its in vitro activity on blood clot. J. Biol. Chem..

[60] Lateef A., Adelere I.A., Kana E.B.G. (2015). *Bacillus safensis* LAU 13: A new source of keratinase and its multi-functional biocatalytic applications. Biotechnol. Biotec. Equip..

[61] Mitsuiki S., Ichikawa M., Oka T., Sakai M., Moriyama Y., Sameshima Y., Goto M., Furukawa K. (2004). Molecular characterization of a keratinolytic enzyme from an alkaliphilic *Nocardiopsis* sp. TOA-1. Enzyme Microb. Technol..

[62] Kim H.C., Choi B.S., Sapkota K., Kim S., Lee H.J., Yoo J.C., Kim S.J. (2011). Purification and characterization of a novel, highly potent fibrinolytic enzyme from *Paecilomyces tenuipes*. Process. Biochem..

[63] Liu X., Kopparapu N.K., Zheng H., Katrolia P., Deng Y., Zheng X. (2016). Purification and characterization of a fibrinolytic enzyme from the food-grade fungus, *Neurospora sitophila*. J. Mol. Catal. B Enzym..

[64] Devaraj Y., Rajender S.K., Halami P.M. (2018). Purification and characterization of fibrinolytic protease from *Bacillus amyloliquefaciens* MCC2606 and analysis of fibrin degradation product by MS/MS. Prep. Biochem. Biotechnol..

[65] Ju X., Cao X., Sun Y., Wang Z., Cao C., Liu J., Jiang J. (2012). Purification and characterization of a fibrinolytic enzyme from *Streptomyces* sp. XZNUM 00004. World J. Microbiol. Biotechnol..

[66] Rashad M.M., Mahmoud A.E., Al-Kashef A.S., Nooman M.U. (2012). Purification and characterization of a novel fibrinolytic enzyme by *Candida guilliermondii* grown on sunflower oil cake. JASR.

[67] Cha W.S., Park S.S., Kim S.J., Choi D. (2010). Biochemical and enzymatic properties of a fibrinolytic enzyme from *Pleurotus eryngii* cultivated under solid-state conditions using corn cob. Bioresour. Technol..

[68] Nascimento T.P., Sales A.E., Porto T.S., Costa R.M.P.B., Breydo L., Uversky V.N., Porto A.L.F., Converti A. (2017). Purification, biochemical, and structural characterization of a novel fibrinolytic enzyme from *Mucor subtilissimus* UCP 1262. Bioproc. Biosyst. Eng..

[69] Afifah D.N., Sulchan M., Syah D. (2014). Purification and characterization of a fibrinolytic enzyme from *Bacillus pumilus* 2. g isolated from Gembus, an Indonesian fermented food. Prev. Nutr. Food Sci..

[70] Esakkiraj P., Meleppat B., Lakra A.K., Ayyannaa R., Arul V. (2016). Cloning, expression, characterization and application of protease produced by *Bacillus cereus* PMW8. RSC Adv..

[71] Kasana R.C., Yadav S.K. (2007). Isolation of a psychrotrophic *Exiguobacterium* sp. SKPB5 (MTCC 7803) and characterization of its alkaline protease. Curr. Microbiol..

[72] Beg Q.K., Gupta R. (2003). Purification and characterization of an oxidation-stable, thiol-dependent serine alkaline protease from *Bacillus mojavensis*. Enzyme Microb. Technol..

[73] Joshi S., Satyanarayana T. (2013). Characteristics and applications of a recombinant alkaline serine protease from a novel bacterium *Bacillus lehensis*. Bioresour. Technol..

[74] Sinha R., Khare S.K. (2013). Characterization of detergent compatible protease of a halophilic *Bacillus* sp. EMB9: Differential role of metal ions in stability and activity. Bioresour. Technol..

[75] Sari E., Logoglu E., Oktemer A. (2015). Purification and characterization of organic solvent stable serine alkaline protease from newly isolated *Bacillus circulans* M34. Biomed. Chromatogr..

[76] Hassanein W.A., Kotb E., Awny N.M., El-Zawahry Y.A. (2011). Fibrinolysis and anticoagulant potential of a metallo protease produced by *Bacillus subtilis* K42. J. Biosci..

[77] Lu C.L., Chen S.N. (2010). Magnesium Enhanced Fibrinolytic Activity of Protease from *Schizophyllum commune*. Taiwania.

[78] Yogesh D., Halami P.M. (2017). Fibrinolytic enzymes of *Bacillus* spp.: An overview. Int. Food Res. J..

[79] Lucy J., Raharjo P.F., Elvina E., Florencia L., Susanti A.I., Pinontoan R. (2019). Clot Lysis Activity of *Bacillus subtilis G8* Isolated from Japanese Fermented Natto Soybeans. Appl. Food Biotechnol..

